# Maternal serum CFHR4 protein as a potential non-invasive marker of ventricular septal defects in offspring: evidence from a comparative proteomics study

**DOI:** 10.1186/s12014-022-09356-y

**Published:** 2022-05-19

**Authors:** Jing He, Liang Xie, Li Yu, Lijun Liu, Hong Xu, Tao Wang, Yuyang Gao, Xuedong Wang, You Duan, Hanmin Liu, Li Dai

**Affiliations:** 1 Department of Pediatric Pulmonology and Immunology, West China Second University Hospital, Sichuan University, 610041 Chengdu, China; 2 Department of Pediatrics, Chengdu Fifth People’s Hospital, Chengdu, 610041 China; 3 The Joint Laboratory for Lung Development and Related Diseases of West China Second University Hospital, Sichuan University and School of Life Sciences of Fudan University, West China Institute of Women and Children’s Health, West China Second University Hospital, Sichuan University, Chengdu, China; 4 Key Laboratory of Birth Defects and Related Diseases of Women and Children (Sichuan University), Ministry of Education, Chengdu, 610041 China; 5 National Center for Birth Defects Monitoring, West China Second University Hospital, Sichuan University, Chengdu, 610041 China; 6 Med-X Center for Informatics, Sichuan University, Chengdu, 610041 China; 7 NHC Key Laboratory of Chronobiology (Sichuan University), Chengdu, China; 8 Sichuan Birth Defects Clinical Research Center, West China Second University Hospital, Sichuan University, Chengdu, China

**Keywords:** Ventricular septal defect, Case–control, Proteomics, Differentially expressed protein, Maternal serum biomarker

## Abstract

**Background:**

Despite advances in diagnosis of congenital heart defects, there is no non-invasive biomarker clinically available for the early detection of fetal ventricular septal defects (VSD).

**Methods:**

This study was to profile differentially expressed proteins (DEP) in the first trimester maternal plasma samples that were collected in the 12th–14th week of gestation and identify potential biomarkers for VSD. Maternal plasma samples of ten case–control pairs of women (who had given birth to an isolated VSD infant or not) were selected from a birth cohort biospecimen bank for identifying DEPs by using high-performance liquid chromatography-tandem mass spectrometry-based comparative proteomics.

**Results:**

There were 35 proteins with significantly different levels between cases and controls, including 9 upregulated and 26 downregulated proteins. With Gene Ontology, Kyoto Encyclopedia of Genes and Genomes pathway enrichment, and protein–protein interaction analyses, most of the DEPs were clustered in pathways related to B cell-mediated immune responses, complement activation, and phagocytosis. Three DEPs were validated using enzyme-linked immunosorbent assay in another set of samples consisting of 31 cases and 33 controls. And CFHR4, a key regulator in complement cascades, was found to be significantly upregulated in cases as compared to controls.

**Conclusions:**

Subsequent logistic regression and receiver operating characteristic analysis suggested maternal serum CFHR4 as a promising biomarker of fetal VSD. Further studies are warranted to verify the findings.

**Supplementary Information:**

The online version contains supplementary material available at 10.1186/s12014-022-09356-y.

## Background

Ventricular septal defects (VSD) are the most common congenital heart diseases (CHD) in human, accounting for 20%–40% of all cardiac anomalies [1–4]. VSD can occur alone or in association with other cardiac or non-cardiac malformations with a prevalence of 1.3–6.5 per 1000 births [1, 4]. The clinical manifestation and prognosis of VSD depend on size and anatomical associations of the anomaly, patient's age, and medical interventions, etc. According to literature, 11%–71% of VSDs can heal spontaneously [3], but approximately 5% of affected infants may die in the neonatal period [5]. Studies on protein biomarkers in maternal blood samples can offer a non-invasive diagnosis of fetal VSD, and provide valuable insights into the molecular mechanisms of this disorder.

Proteomic-based techniques have been used to explore biomarkers in maternal peripheral blood for several fetal birth defects, including Down syndrome, neural tube defects, and CHDs [6–10]. Zhang et al. [8] found that maternal serum gelsolin could be a diagnostic marker for fetal CHDs, while another study by Chen et al. [10] suggested a possible role of cytoskeleton proteins. The inconsistency of results between different studies may be due to heterogeneities in study design, bio-sample sources, CHD subtypes, life styles, or other maternal characteristics. Currently, there are no clinically available non-invasive biomarkers for the prenatal diagnosis of CHDs. Based on maternal plasma specimens obtained from an on-going pregnancy-birth cohort, we conducted a case–control study to profile differentially expressed proteins (DEP) with fetal isolated VSD and to identify potential biomarkers for early diagnosis of this anomaly.

## Methods

### Study subjects

The subjects and samples used in this study were retrospectively selected from a pregnancy-birth cohort initiated by the West China Second University Hospital of Sichuan University [11]. From participants recruited between June 2016 and January 2019, 10 pregnant women who lived in the Chengdu area, had no complications of pregnancy (diabetes, pre-eclampsia and eclampsia), and delivered a singleton with isolated VSD, were selected into the case group. All the VSDs were diagnosed in the second trimester and confirmed after birth, including 7 muscular and 2 perimembranous VSDs, and one muscular case in combination with peri-membranous defect. The control group was comprised of 10 women who delivered a full-term, healthy newborn during the same period, matching on maternal age, infant sex, and gestational age at birth. The peripheral blood samples of these women were collected at the 12th–14th weeks of gestation. For each blood sample, plasma was aliquoted in sterile vacutainers and stored in a refrigerator at − 80 ℃ for proteomics profiling. Next, another set of plasma samples including 31 isolated cases and 33 controls were used to verify the results of proteomics analysis. The study was approved by the Ethics Committee of West China Second University Hospital of Sichuan University (2016-056), and all women participating in the study signed the informed consent form.

### Proteomics analysis

In the discovery phase of this study, plasma samples from ten pairs of case–control women were analyzed by the high-performance liquid chromatography-tandem mass spectrometry (HPLC–MS/MS). Firstly, the cellular debris of 100 μL plasma sample was removed by centrifugation at 12,000*g* at 4 °C for 10 min. The supernatant was transferred to a new centrifuge tube. And the protein concentration was measured with BCA kit (Beyotime P0011, China). Secondly, the solution containing 200 μg protein was reduced with 5 mM dithiothreitol (Sigma, USA) for 30 min at 56 °C and alkylated with 11 mM iodoacetamide (Sigma, USA) for 15 min at room temperature in darkness. Then the protein sample was diluted by adding 100 mM TEAB (Sigma, USA) to urea concentration less than 2 M. To obtain the peptide fragments, trypsin was added at a mass ratio of 1:50 (trypsin-to-protein) for the first digestion overnight and at 1:100 mass ratio for a second digestion for 4 h. Thirdly, tryptic peptides were fractionated into fractions by high-pH (pH 9.0) reversed-phase HPLC using Agilent 300 Extend C18 column (L × I.D. 250 mm × 4.6 mm, 5 μm particle size), with a linear gradient of 8–32% acetonitrile buffer, at a flow rate of 1 mL/min for 60 min into 60 fractions. Mobile phase A (5% acetonitrile in 95% water pH 9) and mobile phase B (5% water in 95% acetonitrile pH 9) was used in this step. Peptide fractions were collected and combined into 48 fractions with the addition of 0.15 μL premixed 10 × indexed Retention Time (iRT) reagent (Biognosys, Switzerland), lyophilised and stored at − 80 °C until use. In the combination procedure, the first fraction was merged with 49th fraction, the second fraction was merged with 50th fraction, and other fractions (3rd ~ 12th and 51th ~ 60th) were combined with the same manner. Fourthly, the lyophilized fractions were redissolved in mobile phase A (5 μL 0.1% formic acid), 3 μL of which was load on and separated by using ReproSil-Pur C18 column (L × I.D. 150 mm × 75 μm, 1.9 μm particle size, 120 Å pore size, Dr. Maisch, Germany) on an Easy 1200 nano-flow LC system. The gradient was comprised of an increase from 5 to 24% mobile phase B (0.1% formic acid in 90% acetonitrile) over 90 min, 24% to 32% in 24 min and climbing to 80% in 3 min then holding at 80% for the last 3 min, all at a constant flow rate of 450 nL/min on UPLC system. Separated peptides were injected into an NSI ion source for ionization, after which data were collected by Q Exactive™ HF-X mass spectrometry (Thermo Scientific, USA), finally the spectral library was constructed for analysis. In detail, the electrospray voltage applied was 2.0 kV. The parent ions of the peptide fragments and their secondary fragments were detected and graded by high-resolution Orbitrap mass spectrometry (Thermo Scientific, USA). The first-order mass spectrometry was performed with a scanning range of 385–1200 m/z and a resolution of 120,000, followed 70 s-order mass spectrometry analysis under the data independent acquisition (DIA) mode with 27% fragmentation energy and a scan resolution of 15,000. The automatic gain control (ACG) of the second-order mass spectrometry was set to 5E5 and the fixed first mass was fixed at 200 m/z.

DDA mode was used to build reference spectral library before DIA data acquisition. The DDA data of all 48 fractions was used to searching against SwissProt_Human (20387 sequences) database with Maxquant (v 1.6.6.0) for protein identification [12]. The enzyme digestion method was set to Trypsin/P, the number of missed cleavage sites was set to 2, and the mass error tolerances of the primary precursor ions were set to 20 ppm and 4.5 ppm for first search and main search, respectively. And the mass error tolerance of the secondary fragment ions was 0.02 Da. Alkylation of cysteine was set as a fixed modification, and the oxidation of methionine protein N-termini was set as a variable modification. The false discovery rate (FDR) for the identification of protein and peptide spectrum matches was set at 1%.

Based on the LC–MS/MS spectral library matched with known peptide identifications, Skyline (v4.1.0) software was used to analysis DIA data for protein quantification [13]. The corresponding spectral library was imported into the software and analyzed with corresponding iRT parameters for prediction. The parent ion charge of Transition was set at 2, 3, and 4, and the daughter ion charge was set at 1 and 2. Six ions with the highest intensity were extracted from the spectral library for peptide fragment quantification. DIA data were imported after generating the reverse database, and the mProphet algorithm was used for FDR filtering. The relative quantification results of the proteins were exported for next analysis.

### Bioinformatic analysis

In this study, protein ratio (fold change) > 1.2 or < 0.83 was considered differentially expressed between cases and controls. Protein set enrichment analysis was performed using Gene Ontology functional database (GO) to explore the biological effects of DEPs, and protein–protein interaction was created and visualized by the STRING database tools (https://www.string-db.org, version 10.5) [14]. Potential pathways of genes that encode DEPs were analyzed in the Kyoto Encyclopedia of Genes and Genomes (KEGG) database. Functional enrichment analysis was performed on the DEPs using the cluster Profiler package (v4.0.5) [15].

### Validation study

Enzyme-linked immunosorbent assay (ELISA) was performed to validate selected DEPs in another set of case–control samples. Three candidate proteins with commercially available kits were selected for ELISA because they were highly differentially expressed (absolute fold change close to or greater than 2), including mannose-binding lectin 2 (MBL2, Abonova, Taiwan), complement factor H-related protein 4 (CFHR4, Novus Biologicals, USA) and immunoglobulin kappa variable 4-1(IGKV4-1, Biorbyt, UK). The experiments were conducted according to the manufacturer’s manuals. Briefly, diluted plasma samples (100 µL per well) were each added into duplicate wells together with 100 µL biotinylated detection Ab working solution per well in a 96-well plate. Two wells of blank controls, negative controls, and positive controls were included on each plate. After 2-h incubation at 37 ℃ on a microplate shaker rotating at 100 rpm, the plate was washed. Then 100 μL of HRP Conjugate working solution were added to each well and incubated for 30 min at 37 °C. After washing, color development was mediated by the addition of 90 µL tetramethylbenzidine substrate solution to each well and incubation at 37℃ for 15 min. Following stopping the reaction, optical absorbance of each microwell was immediately measured at 450 nm by using a spectro-photometer (Infinite M200, Tecan Trading AG, Switzerland). The concentration of each analyte was determined by referring to a standard curve generated simultaneously on the same plate.

### Statistical analysis

The maternal serum protein levels between cases and controls were examined by using two-sided t-tests. Logistic regression and receiver operating characteristic (ROC) curve analysis were adopted to assess the diagnostic performance of candidate proteins validated by ELISA. The statistical significance level for α was set at 0.05.

## Results

### General characteristics of the study subjects

Table 1 shows the maternal and infant characteristics by case–control status. We compared the distribution of maternal age, periconceptional folic acid supplementation, history of adverse pregnancy outcomes (spontaneous abortion, stillbirth, and termination of pregnancy), caesarean delivery, infant sex, birth weight, length and gestational age at birth between the cases and controls. No significant difference was found in any of aforementioned characteristics between the cases and controls for proteomic discovery. In the case–control set for validation, more case women had the history of adverse pregnancy outcomes (58.1% vs 24.2%) and adopted cesarean delivery (71.0% vs 45.5%) than control women. And the birthweight of VSD infants was significantly lighter than that of healthy newborns (3108 g vs 3350 g).

**Table 1:** General characteristics of the study subjects

Characteristics	For discovery			For validation		
	Cases	Controls	*p*	Cases	Controls	*p*
N	10	10		31	33	
Maternal age (years)	29 (25–33)	29.5 (25–34)	NS	31.9 (25–38)	31.4 (26–36)	NS
Maternal BMI	20.5 (19.0–26.5)	20.9 (17.8–25.5)	NS	21.6 (15.6–27.2)	21.7 (18.0–28.6)	NS
Periconceptional folic acid supplementation	6 (60.0%)	4 (40.0%)	NS	21 (67.7%)	16 (48.5%)	NS
History of adverse pregnancy outcomes*	2 (20.0%)	3 (30.0%)	NS	18 (58.1%)	8 (24.2%)	0.006
Caesarean delivery	7 (70.0%)	4 (40.0%)	NS	22 (71.0%)	15 (45.5%)	0.039
Sex of infant (Female/male)	5/5	5/5	NS	13/18	15/18	NS
Gestational age (days)	277.6 (271–289)	276.7 (262–290)	NS	275.3 (243–301)	276.8 (259–294)	NS
Birth weight of infant (g)	3547 (3100–3800)	3304.4 (2500–3700)	NS	3108.2 (1640–4040)	3350 (2600–3980)	0.03
Length of infant (cm)	50.9 (48–53)	49.9 (46–51)	NS	49.3 (43–54)	49.9 (46–53)	NS

NS: not statistically significant

^*^Adverse pregnancy outcomes included spontaneous abortion, induced abortion, and stillbirth. In validation set, there were 7 case women with history of spontaneous abortion, 10 with history of induced abortion, and 1 with history of stillbirth, respectively; there were 2 control women with history of spontaneous abortion, 5 with history of induced abortion, and 1 with stillbirth, respectively

### Proteomics analysis

**Fig. 1:**
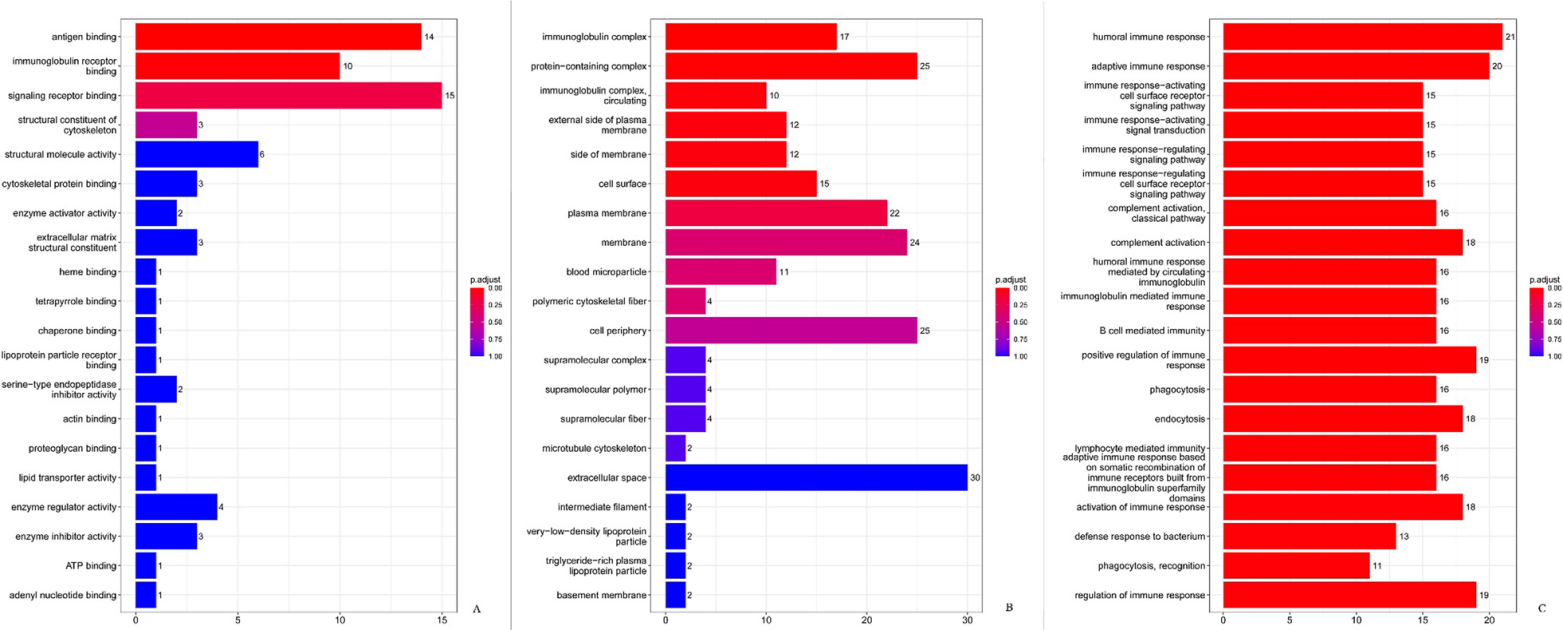
Differentially expressed proteins under each GO category. **A** Molecular function; **B** cellular component; **C** biological process

A total of 7942 peptide fragments were identified by using HPLC–MS/MS, among which 787 proteins were matched with known proteins, and relative expression information of 497 proteins were obtained. There were 35 proteins expressed differentially between cases and controls (absolute fold change ≥ 1.2), with 9 upregulated and 26 downregulated in cases compared with controls. GO functional analysis indicated that these DEPs were mainly clustered in the molecular activities of binding, catalysis, regulation, and transduction, involved in the cellular processes of biological regulation, stimulus response, metabolism, development, and the immune system (Fig. 1). In terms of biological processes, these proteins were enriched in the immunoglobulin-regulated immune response, B cell-mediated immunity, and phagocytosis. KEGG analysis revealed that these DEPs were enriched in Fc-gamma receptor, Fc-epsilon receptor, B cell receptor, phospholipase D, calcium signaling pathways, etc. (Additional file 1: Table S1).

Five proteins were considered highly differentially expressed (absolute fold change ≥ 2.0), including the upregulated keratin 5 (KRT5) and complement factor H-related protein 4 (CFHR4), and the downregulated mannose-binding lectin 2 (MBL2), immunoglobulin light chain variable region 3-25 (IGLV3-25), and immunoglobulin heavy chain variable region 1-24 (IGHV1-24) (Additional file 1: Table S1). IGLV3-25, IGHV1-24, and MBL2 were involved in various immunoglobulin-mediated immune responses, such as the B cell-mediated immune response, phagocytosis, receptor-mediated endocytosis, and response to coronavirus, while CFHR4 was mainly implicated in complement activation pathway (Additional file 1: Table S1 and Additional file 2: Fig. S1).

We mapped 16 out of 35 DEPs onto the human protein–protein interaction network with the confidence cutoff of 0.7 to retrieve known interaction links. By comparing the proteins against STRING (v.10.5), the 16 DEPs accounted for a large proportion in the network. Beta-2-glycoprotein 1 (APOH), fibrinogen alpha chain (FGA), and fibrinogen beta chain (FGB) had 9 documented interactions, and basement membrane-specific heparan sulfate proteoglycan core protein (HSPG2) had 8 known interactions in this network. Other highly differentially proteins such as CHFR4, MBL2, and KRT5 had 1, 4, and 2 known interactions, respectively. (Additional file 3: Fig. S2 and Additional file 1: Table S2).

### Validation of candidate proteins

**Fig. 2:**
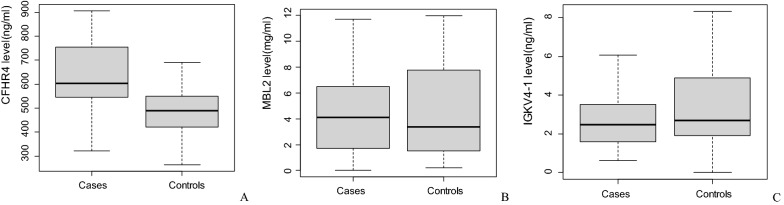
Box plot of selected protein markers in first trimester maternal plasma by case–control status. **A** CFHR4; **B** MBL2; **C** IGKV4-1

**Fig. 3:**
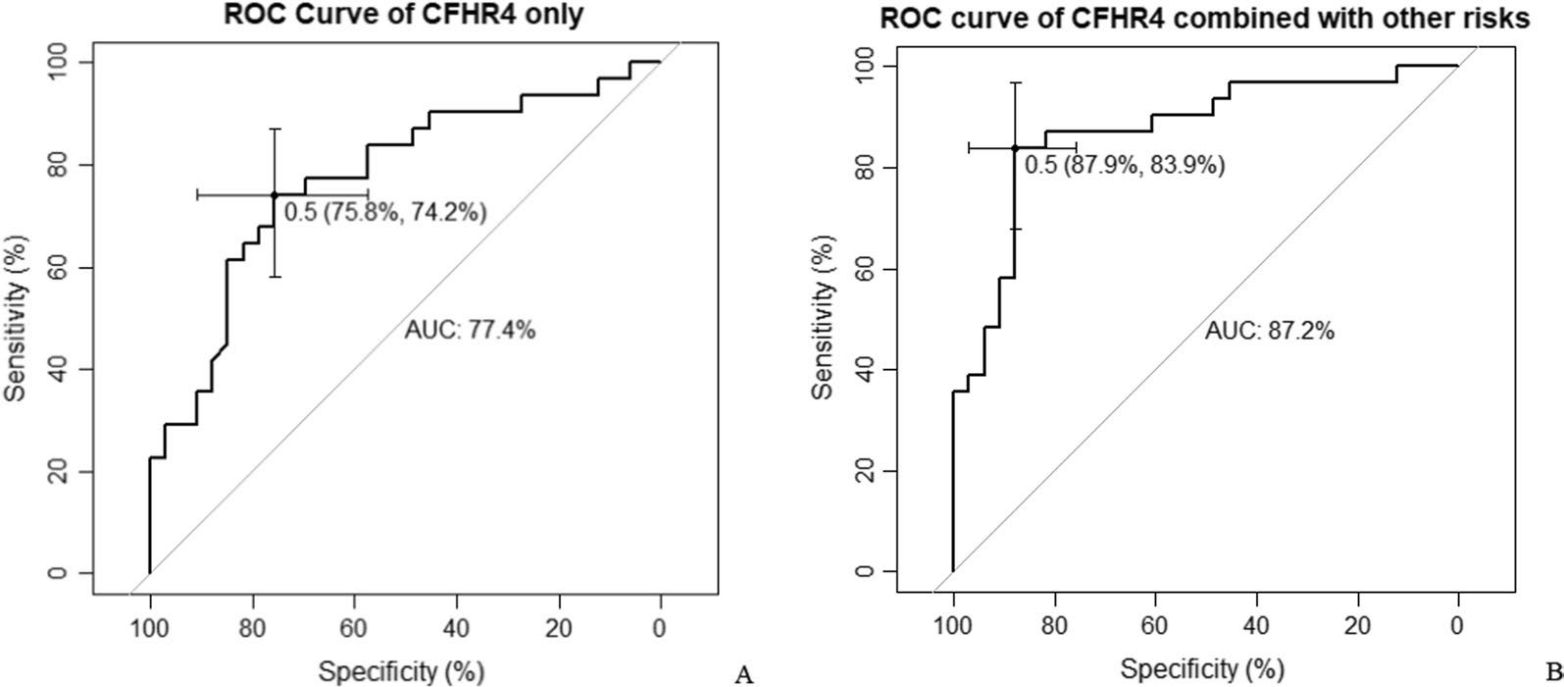
The receiver operating characteristic curves showing the performance of CHFR4. **A** The model included CFHR4 only; **B** adjusted for maternal body mass index during the first trimester, history of adverse pregnancy outcomes, and periconceptional folic acid supplementation

ELISA was developed to validate MBL2, CFHR4 and IGKV4-1 in additional case–control samples. As shown in Table 2 and Fig. 2, the maternal serum level of CFHR4 was significantly higher in cases than that in controls. Further binary logistic regression and ROC analysis revealed that CFHR4 could be a potential predictive marker for fetal VSD, with an uncorrected *p*-value of 0.0001 and an area under the curve of 0.774 (95% CI 0.656–0.891) (Fig. 3A). When maternal body mass index during the first trimester, history of adverse pregnancy outcomes and periconceptional folic acid supplementation were included into the modelling analysis, CFHR4 still contributed significantly, with an area under curve (AUC) of 0.872 (95% CI 0.782–0.962) (Fig. 3B). Maternal age, gestational age and infant sex were omitted from the analysis since the cases and controls were matched on these factors. Detailed information on multivariate logistic regression model was presented in Additional file 1: Table S3.

**Table 2:** Protein serum levels measured by ELISA in the validation

Protein	Cases (mean ± SD)	Controls (mean ± SD)	*p* value
CFHR4 (ng/mL)	631.59 ± 151.78	494.27 ± 112.06	0.0001
MBL2 (mg/mL)	4.69 ± 3.38	4.68 ± 3.66	NS
IGKV4-1 (ng/mL)	3.07 ± 2.16	6.66 ± 10.46	0.09

NS: not statistically significant

## Discussion

The present study identified 35 DEPs in first trimester plasma from the case and control pregnant women using HPLC–MS/MS proteomic analysis. ELISA validation and subsequent analysis confirmed significantly up-regulated CFHR4 in cases, suggesting maternal CFHR4 as a potential non-invasive biomarker of fetal VSD. The findings could be of value in understanding the pathogenesis and early detection of this congenital disorder.

The DEPs found in our study are mainly associated with immune responses and complement cascades. Totally, 20 DEPs are subunits of immunoglobins that were downregulated in the cases. Among the five highly differentially expressed proteins, IGLV3-25 and IGHV1-24 are implicated in cell immunoglobulin-mediated immune responses, while CFHR4 and MBL2 play important roles in activating complement pathways. MBL2, a calcium-dependent factor, can initiate the complement lectin pathway to induce lysis and opsonophagocytosis by binding to mannose and other oligosaccharide structures on the surface of pathogens, apoptotic cells, and some tumor cells [16, 17]. Insufficiency of MBL2 or other innate immune components has been reported to be associated with premature birth, low birth weight, and miscarriage [18, 19]. Stimulating cellular immune function of pregnant mice could reduce the risk of cleft palate, limb deformities, neural tube defects, and eye malformations in offspring due to teratogens or maternal diabetes [20–23]. In this study, the downregulated immunoglobulins and MBL2, and upregulated CFHR4 in cases may represent a disruption or imbalance of maternal immune homeostasis, suggesting the importance of maternal immunity to fetal heart development. A rat model study has shown that maternal anti-cardiac-myosin autoantibodies could enter fetal circulation through placental passage and increase the risk of fetal structural heart defects [24]. Researchers have noticed the associations of immune and inflammatory associations for a long time, and emphasized the significance of studying CHD from an immunological perspective [25]. The underlying mechanisms for these associations remain unclear. One possible explanation is that activating maternal cellular immunity may improve the synthesis and release of immunoglobulins, therefore mediating the effect of in utero exposure to teratogens through complex interactions with the fetus or placenta [23].

Another highly DEP, KRT5, a type II cytokeratin specifically expressed in human epidermis, is involved in the formation of cytoskeleton [26]. The reorganization of cytoskeleton is necessary for the development of cardiac cells and heart morphogenesis [27]. Several cytoskeleton proteins have been linked to CHD or related malformations, such as the association of decreased maternal plasma transthyretin with fetal Down syndrome accompanied by CHDs [28, 29], and four cytoskeleton proteins in maternal plasma as potential biomarkers for detecting CHDs in offspring. However, no direct evidence in favor of a role of KRT5 in fetal cardiac development. The associations of potential DEPs with CHDs and the underlying mechanisms still need more investigations.

ELISA was used to validate three candidate DEPs (IGKV4-1, MBL2 and CFHR4) in additional case–control set. Consistent with proteomics analysis, maternal plasma CHFR4 was significantly higher in cases than in controls, whereas no significant difference in MBL2 and IGKV4-1 was found. CFHR4, mainly produced in hepatocytes [30], regulates the activation of the complement system by competing with complement factor H (CFH) for the binding to complement C3b, C-reactive protein (CRP) and human cell surfaces [31]. CFH is a key regulator of the alternative activation pathway of human complement system, orchestrating complement activation to foreign cells by specifically binding to and inhibiting complement on cells. As a competitor, the binding of CFHR4 instead of CFH might serve to fine-tune the inhibition by CFH on cell surfaces where balanced complement activation is crucial for the clearance of necrotic and apoptotic cells. Excessive CFHR4 enhances the cofactor activity of CFH, and correlates to certain inflammatory conditions [32]. A study reported that CFHR4 recruited CRP to necrotic cells in vitro*,* allowing more complement activation to occur [31]. In this study, a higher level of CFHR4 in cases suggested that imbalanced complement activation or detrimental inflammatory response might have occurred, thus increased the risk of fetal VSD introduced by pathogens. Genetic variations in CFHR4 gene have been linked to age-related macular degeneration, systemic lupus erythematosus, and atypical hemolytic uremic syndrome [33]. And maternal systemic lupus erythematosus is a known risk factor of congenital heart defects [25]. It is still unclear what the role of CFHR4 is within the complement system as well as in fetal cardiac development. In-depth exploration on the relationship between congenital malformations and changes in maternal plasma CFHR4 or other related proteins at different stages of pregnancy will help to understand the role of these molecules in fetal heart development and CHDs.

With ELISA validation and subsequent logistic regression analysis, first trimester maternal plasma CFHR4 was confirmed as a biomarker of fetal VSD with an AUC of 0.77. Limited studies have applied quantitative proteomic techniques on exploring maternal serum biomarkers for CHDs. Zhang et al. found that gelsolin in maternal serum samples collected in the 14–18 weeks of gestation could be a biomarker of conotruncal heart defects [8]. Another study identified that a panel composed of four cytoskeletal proteins (LMNA, FLNA, TPM4, and ACTG1) could discriminate CHD-pregnancies from normal ones with an AUC of 0.938 by detecting the protein levels in maternal serum samples collected at 22 to 26 weeks of gestation [10]. A most recent study published in late 2021 suggested that the lactoferrin in first trimester maternal serum-derived exosomes may be a potential non-invasive biomarker of fetal isolated VSD [34]. At present, ultrasound imaging is the major tool for prenatal diagnosis of CHDs. The accuracy highly depends on the skills of the operators, status of equipment and other factors such as fetal position, overweight or obesity of pregnant women, etc. There is an increasing demand for the development of non-invasive techniques for the early screening of CHDs. In this study, proteomic profiling enabled us to detect a number of differentially expressed proteins between cases and controls. The addition of more known risk factors to the model greatly improved the ability to discriminate VSD cases from normal pregnancies, which seems attractive for clinical applications and future studies.

The current study has several strengths. Firstly, the use of HPLC–MS/MS proteomic technology followed by ELISA validation experiments ensure the stability and reproducibility of the results [35]. Secondly, the plasma samples for omics were carefully selected and matched on maternal age, gestational age and infant sex. The samples and clinical data were obtained from well-established pregnancy-birth cohort, which reduces the effect of information bias and increases the detectability of differentially expressed proteins. Thirdly, multivariate logistic regression and ROC curve analysis further highlight the potential of first trimester maternal plasma CFHR4 in predicting fetal VSD. However, this study is preliminary and its sample-size is relatively small. Several potential DEPs could not be further examined in the case–control series due to the unavailability of ELISA kits. Therefore, future researches are warranted to verify the findings and explore the clinical utility of potential protein markers.

## Conclusion

In conclusion, the identified DEPs in first trimester maternal plasma show potentials to be biomarkers for early prenatal screening of VSD. Specifically, maternal CFHR4 may be an independent predictor, and the discriminative power will be improved when using in combination with other risk factors.

## Supplementary Information

**Additional file 1: Table S1**. Functional GO analysis of 35 differentially expressed proteins between cases and controls.** Table S2. **The results table of protein–protein interaction analysis on selected differentially expressed proteins.** Table S3. **Multivariate logistic regression analysis on the association of maternal plasma CFHR4 with fetal VSD.

**Additional file 2**: **Fig. S1. **KEGG pathway enrichment bubble plot of differentially expressed proteins.

**Additional file 3: Fig. S2. **The protein–protein interaction network of selected differentially expressed proteins.

## Data Availability

Not applicable.

## Abbreviations

CFHR4: Complement factor H-related-4
VSD: Ventricular septal defects
CFHR: Complement factor H-related proteins
CHD: Congenital heart diseases
DEP: Differentially expressed protein
DIA: Data independent acquisition
FDR: False discovery rate
HPLC: High-performance liquid chromatography
HPLC–MS/MS: High-performance liquid chromatography–tandem mass spectrometry
LC–MS/MS: Liquid chromatography–tandem mass spectroscopy
ELISA: Enzyme-linked immunosorbent assay
GO: Gene Ontology
KEGG: Kyoto encyclopedia of genes and genomes
PPI: Protein–protein interaction
ROC: Receiver operating characteristic curve
AUC: Area under curve
KRT5: Keratin 5
IGHV1-24: Immunoglobulin heavy chain variable region 1-24
IGLV3-25: Immunoglobulin light chain variable region 3-25
MBL2: Mannose-binding lectin 2
APOH: Beta-2-glycoprotein 1
FGA: Fibrinogen alpha chain
FGB: Fibrinogen beta chain
HSPG2: Basement membrane-specific heparan sulfate proteoglycan core protein
CFH: Complement factor H
CRP: C-reactive protein
LMNA: Isoform A of Prelamin-A/C
FLNA: Filamin A
TPM4: Tropomyosin alpha-4 chain
ACTG1: Actin Gamma 1
