# Supplementary material for: Maternal serum CFHR4 protein as a potential non-invasive marker of ventricular septal defects in offspring: evidence from a comparative proteomics study
Source: Clin Proteomics. 2022 May 19;19:17. doi: 10.1186/s12014-022-09356-y (PMC9117979; doi:10.1186/s12014-022-09356-y)
Supplement: Supplementary file 1 — Additional file 1: Table S1. Functional GO analysis of 35 differentially expressed proteins between cases and controls. Table S2. The results table of protein–protein interaction analysis on selected differentially expressed proteins. Table S3. Multivariate logistic regression analysis on the association of maternal plasma CFHR4 with fetal VSD. [file 12014_2022_9356_MOESM1_ESM.docx]

**Table S1 Functional GO analysis of 35 differentially expressed proteins between cases and controls.**

| **Protein Accession** | **Gene*** | **Case/Control Log2FC** | **Case/Control ratio** | ***p* value** | **Molecular Function** | **Cellular Component** | **Biological Process** |
| --- | --- | --- | --- | --- | --- | --- | --- |
| P01717 | *IGLV3-25* | -1.29 | 0.41 | 0.0003 | serine-type endopeptidase activity | Vesicle; extracellular space; plasma membrane | complement activation, classical pathway; Fc-gamma receptor signaling pathway involved in phagocytosis; receptor-mediated endocytosis; proteolysis |
| Q92496 | *CFHR4* | 1.284 | 2.44 | 0.0072 | lipid transporter activity | extracellular region | lipid transport |
| P13647 | *KRT5* | 1.271 | 2.41 | 0.0069 | scaffold protein binding;  structural constituent of cytoskeleton | extracellular exosome; keratin filament | hemidesmosome assembly; cornification |
| P11226 | *MBL2* | -1.225 | 0.43 | 0.0059 | mannose binding; calcium ion binding; receptor binding; calcium-dependent protein binding; serine-type endopeptidase activity | cell surface; extracellular space; collagen trimer | modification by host of symbiont morphology or physiology; disruption of cells of other organism involved in symbiotic interaction; killing by host of symbiont cells; positive regulation of endocytosis; positive regulation of phagocytosis; defense response to Gram-positive bacterium; complement activation, lectin pathway; acute-phase response; complement activation, classical pathway; protein activation cascade; proteolysis; opsonization |
| A0A0C4DH33 | *IGHV1-24* | -1.096 | 0.47 | 0.0034 | immunoglobulin receptor binding; antigen binding | immunoglobulin complex, circulating; blood microparticle; external side of plasma membrane | B cell receptor signaling pathway; phagocytosis, engulfment; phagocytosis, recognition; positive regulation of B cell activation; complement activation, classical pathway; innate immune response; defense response to bacterium |
| P0DP03 | *IGHV3-30-5* | -0.941 | 0.52 | 0.0193 | protein binding | - | - |
| P01859 | *IGHG2* | -0.92 | 0.53 | 0.0091 | serine-type endopeptidase activity; immunoglobulin receptor binding; antigen binding | external side of plasma membrane; blood microparticle; immunoglobulin complex, circulating; extracellular exosome | innate immune response; defense response to bacterium; protein activation cascade; proteolysis; complement activation, classical pathway; positive regulation of B cell activation; Fc-gamma receptor signaling pathway involved in phagocytosis; B cell receptor signaling pathway |
| P01766 | *IGHV3-13* | -0.876 | 0.54 | 0.0066 | serine-type endopeptidase activity; protein binding; antigen binding | blood microparticle; extracellular exosome; plasma membrane | receptor-mediated endocytosis; Fc-gamma receptor signaling pathway involved in phagocytosis; Fc-epsilon receptor signaling pathway; leukocyte migration; complement activation, classical pathway; protein activation cascade; proteolysis |
| A0A0B4J1U3 | *IGLV1-36* | -0.858 | 0.55 | 0.0085 | protein binding; antigen binding | extracellular space; plasma membrane | immunoglobulin production; adaptive immune response |
| P68133 | *ACTA1* | 0.787 | 1.73 | 0.0191 | ATP binding; ADP binding; myosin binding; structural constituent of cytoskeleton | striated muscle thin filament; lamellipodium; filopodium; cytosol; actin filament; blood microparticle; extracellular exosome; stress fiber | positive regulation of gene expression; response to steroid hormone; response to lithium ion; skeletal muscle thin filament assembly; skeletal muscle fiber development; skeletal muscle fiber adaptation; mesenchyme migration |
| A0A0B4J1V0 | *IGHV3-15* | -0.782 | 0.58 | 0.0007 | immunoglobulin receptor binding; antigen binding | external side of plasma membrane; blood microparticle; immunoglobulin complex, circulating | complement activation, classical pathway; innate immune response; defense response to bacterium; positive regulation of B cell activation; B cell receptor signaling pathway; phagocytosis, engulfment |
| A0A075B6P5 | *IGKV2-28* | -0.737 | 0.60 | 0.0089 | serine-type endopeptidase activity; protein binding; antigen binding | extracellular space; plasma membrane | Fc-gamma receptor signaling pathway involved in phagocytosis; Fc-epsilon receptor signaling pathway; immunoglobulin production; receptor-mediated endocytosis; proteolysis; protein activation cascade; complement activation, classical pathway; leukocyte migration |
| P01743 | *IGHV1-46* | -0.668 | 0.63 | 0.0200 | serine-type endopeptidase activity; antigen binding; protein binding | extracellular region; plasma membrane | Fc-epsilon receptor signaling pathway; Fc-gamma receptor signaling pathway involved in phagocytosis; receptor-mediated endocytosis; leukocyte migration; complement activation, classical pathway; protein activation cascade; proteolysis |
| P98160 | *HSPG2* | 0.611 | 1.53 | 0.0236 | protein C-terminus binding; calcium ion binding | Golgi lumen; lysosomal lumen; extracellular exosome; adherens junction; focal adhesion; basement membrane; plasma membrane | glycosaminoglycan catabolic process; glycosaminoglycan biosynthetic process; angiogenesis; cellular protein metabolic process; retinoid metabolic process; extracellular matrix disassembly |
| P0CG38 | *POTEI* | 0.603 | 1.52 | 0.0099 | protein binding | extracellular exosome | retina homeostasis |
| P06312 | *IGKV4-1* | -0.588 | 0.67 | 0.0148 | serine-type endopeptidase activity; protein binding; antigen binding | plasma membrane; blood microparticle | receptor-mediated endocytosis; Fc-epsilon receptor signaling pathway; Fc-gamma receptor signaling pathway involved in phagocytosis; leukocyte migration; complement activation, classical pathway; immunoglobulin production; proteolysis |
| P01718 | *IGLV3-27* | -0.583 | 0.67 | 0.0475 | serine-type endopeptidase activity; protein binding; antigen binding | extracellular region; plasma membrane | receptor-mediated endocytosis; Fc-gamma receptor signaling pathway involved in phagocytosis; leukocyte migration; complement activation, classical pathway; protein activation cascade; proteolysis |
| P01701 | *IGLV1-51* | -0.527 | 0.69 | 0.0333 | serine-type endopeptidase activity; protein binding; antigen binding | extracellular exosome; plasma membrane | receptor-mediated endocytosis; Fc-gamma receptor signaling pathway involved in phagocytosis; Fc-epsilon receptor signaling pathway; leukocyte migration; protein activation cascade; proteolysis |
| P02671 | *FGA* | 0.52 | 1.43 | 0.0089 | metal ion binding; receptor binding; protein binding, bridging; structural molecule activity | platelet alpha granule lumen; external side of plasma membrane; cytoplasmic region; cell cortex; blood microparticle; fibrinogen complex; extracellular exosome | plasminogen activation; post-translational protein modification; positive regulation of vasoconstriction; blood coagulation, common pathway; fibrinolysis; response to calcium ion; induction of bacterial agglutination; innate immune response; positive regulation of substrate adhesion-dependent cell spreading; toll-like receptor signaling pathway; platelet aggregation; positive regulation of heterotypic cell-cell adhesion; negative regulation of extrinsic apoptotic signaling pathway via death domain receptors; positive regulation of peptide hormone secretion; protein polymerization; extracellular matrix organization; positive regulation of exocytosis; positive regulation of ERK1 and ERK2 cascade; negative regulation of endothelial cell apoptotic process; platelet degranulation |
| A0A0B4J1V1 | *IGHV3-21* | -0.511 | 0.70 | 0.0053 | immunoglobulin receptor binding; antigen binding | immunoglobulin complex, circulating; blood microparticle; external side of plasma membrane | positive regulation of B cell activation; complement activation, classical pathway; innate immune response; defense response to bacterium; B cell receptor signaling pathway; phagocytosis, engulfment; phagocytosis, recognition |
| P01601 | *IGKV1D-16* | -0.505 | 0.70 | 0.0234 | serine-type endopeptidase activity; protein binding; antigen binding | extracellular exosome; plasma membrane | receptor-mediated endocytosis; Fc-epsilon receptor signaling pathway; Fc-gamma receptor signaling pathway involved in phagocytosis; leukocyte migration; complement activation, classical pathway; protein activation cascade; immunoglobulin production; proteolysis |
| P35908 | *KRT2* | 0.505 | 1.42 | 0.0390 | cytoskeletal protein binding; structural constituent of cytoskeleton | extracellular exosome; keratin filament | intermediate filament organization; keratinocyte proliferation; cornification; keratinocyte activation; keratinocyte migration; keratinocyte development |
| A0A0B4J1X5 | *IGHV3-74* | -0.493 | 0.71 | 0.0101 | immunoglobulin receptor binding; antigen binding | immunoglobulin complex, circulating; blood microparticle; external side of plasma membrane | protein activation cascade; defense response to bacterium; positive regulation of B cell activation; complement activation, classical pathway; innate immune response; B cell receptor signaling pathway; phagocytosis, engulfment |
| P02655 | *APOC2* | 0.485 | 1.40 | 0.0215 | protein homo dimerization activity; lipoprotein lipase activator activity; phospholipase binding phospholipase activator activity; lipid binding | spherical high-density lipoprotein particle; low-density lipoprotein particle; intermediate-density lipoprotein particle; very-low-density lipoprotein particle; chylomicron; extracellular exosome; early endosome; cytosol | positive regulation of fatty acid biosynthetic process; retinoid metabolic process; positive regulation of triglyceride catabolic process; negative regulation of very-low-density lipoprotein particle clearance; chylomicron remnant clearance; high-density lipoprotein particle clearance; chylomicron assembly; high-density lipoprotein particle remodeling; chylomicron remodeling; positive regulation of very-low-density lipoprotein particle remodeling; negative regulation of receptor-mediated endocytosis; negative regulation of cholesterol transport; cholesterol efflux; reverse cholesterol transport; phospholipid efflux; cholesterol homeostasis; positive regulation of phospholipase activity; positive regulation of lipoprotein lipase activity |
| Q8TF30 | *WHAMM* | -0.48 | 0.72 | 0.0106 | small GTPase binding; Arp2/3 complex binding; microtubule binding; actin binding | cytoplasmic vesicle membrane; endoplasmic reticulum-Golgi intermediate compartment membrane; microtubule; Golgi membrane; cytosol | actin filament reorganization; Arp2/3 complex-mediated actin nucleation; positive regulation of actin nucleation; ER to Golgi vesicle-mediated transport; plasma membrane tubulation; adherens junction assembly; focal adhesion assembly; lamellipodium assembly; biogenesis; biological regulation |
| P0DP02 | *IGHV3-30-3* | -0.461 | 0.73 | 0.0028 | protein binding | - | - |
| P04196 | *HRG* | -0.457 | 0.73 | 0.0437 | serine-type endopeptidase inhibitor activity; enzyme inhibitor activity; cysteine-type endopeptidase inhibitor activity; zinc ion binding; anion binding; heme binding; immunoglobulin binding; receptor binding; heparan sulfate proteoglycan binding; heparin binding | blood microparticle; plasma membrane; extracellular exosome; platelet alpha granule lumen | positive regulation of immune response to tumor cell; defense response to fungus; antimicrobial humoral immune response mediated by antimicrobial peptide; negative regulation of fibrinolysis; positive regulation of blood vessel remodeling; negative regulation of endothelial cell chemotaxis; negative regulation of blood vessel endothelial cell migration; negative regulation of vascular endothelial growth factor signaling pathway; negative regulation of angiogenesis; heme transport; platelet degranulation; negative regulation of cell growth; regulation of protein complex assembly; negative regulation of lamellipodium assembly; regulation of actin cytoskeleton organization; positive regulation of focal adhesion assembly; negative regulation of cell adhesion mediated by integrin; regulation of peptidyl-tyrosine phosphorylation; positive regulation of apoptotic process; negative regulation of endopeptidase activity |
| P01834 | *IGKC* | -0.434 | 0.74 | 0.0203 | serine-type endopeptidase activity; immunoglobulin receptor binding; antigen binding | external side of plasma membrane; blood microparticle; extracellular exosome; immunoglobulin complex, circulating | defense response to bacterium; innate immune response; positive regulation of B cell activation; Fc-gamma receptor signaling pathway involved in phagocytosis; B cell receptor signaling pathway; complement activation, classical pathway; protein activation cascade; phagocytosis, recognition; phagocytosis, engulfment; receptor-mediated endocytosis; proteolysis; retina homeostasis |
| P04433 | *IGKV3-11* | -0.434 | 0.74 | 0.0132 | serine-type endopeptidase activity; protein binding; antigen binding | plasma membrane; extracellular exosome; blood microparticle | Fc-gamma receptor signaling pathway involved in phagocytosis; Fc-epsilon receptor signaling pathway; complement activation, classical pathway; leukocyte migration; protein activation cascade; receptor-mediated endocytosis; proteolysis |
| P02675 | *FGB* | 0.43 | 1.35 | 0.0292 | protein binding, bridging; chaperone binding; receptor binding; structural molecule activity | platelet alpha granule lumen; cytoplasmic region; cell cortex; external side of plasma membrane; extracellular exosome; fibrinogen complex; blood microparticle | induction of bacterial agglutination; innate immune response; response to calcium ion; cellular response to interleukin-1; cellular response to leptin stimulus; fibrinolysis; blood coagulation, fibrin clot formation; positive regulation of ERK1 and ERK2 cascade; negative regulation of endothelial cell apoptotic process; positive regulation of vasoconstriction; plasminogen activation; negative regulation of extrinsic apoptotic signaling pathway via death domain receptors; positive regulation of substrate adhesion-dependent cell spreading; protein polymerization; extracellular matrix organization; positive regulation of heterotypic cell-cell adhesion; positive regulation of peptide hormone secretion |
| P01780 | *IGHV3-7* | -0.417 | 0.75 | 0.0292 | serine-type endopeptidase activity; protein binding antigen binding | extracellular exosome; plasma membrane | receptor-mediated endocytosis; Fc-epsilon receptor signaling pathway; Fc-gamma receptor signaling pathway involved in phagocytosis; leukocyte migration; complement activation, classical pathway; protein activation cascade; proteolysis |
| A0A075B6J9 | *IGLV2-18* | -0.414 | 0.75 | 0.0158 | protein binding; antigen binding | extracellular space; plasma membrane | immunoglobulin production; adaptive immune response |
| P36955 | *SERPINF1* | -0.342 | 0.79 | 0.0008 | enzyme inhibitor activity; serine-type endopeptidase inhibitor activity | perinuclear region of cytoplasm; axon hillock; basement membrane; melanosome; extracellular exosome | negative regulation of angiogenesis; retina development in camera-type eye; positive regulation of neurogenesis; negative regulation of endothelial cell migration; negative regulation of epithelial cell proliferation involved in prostate gland development; short-term memory; positive regulation of neuron projection development; negative regulation of neuron death; cellular response to glucose stimulus; cellular response to cobalt ion; cellular response to retinoic acid; cellular response to dexamethasone stimulus; negative regulation of gene expression |
| Q03591 | *CFHR1* | -0.331 | 0.79 | 0.0083 | - | extracellular exosome; blood microparticle | protein activation cascade; complement activation |
| P02749 | *APOH* | -0.272 | 0.83 | 0.0193 | heparin binding; identical protein binding; phospholipid binding; anion binding; lipoprotein lipase activator activity | platelet dense granule lumen; high-density lipoprotein particle; extracellular exosome; chylomicron; very-low-density lipoprotein particle | plasminogen activation; platelet degranulation; triglyceride metabolic process; negative regulation of smooth muscle cell apoptotic process; negative regulation of endothelial cell proliferation; negative regulation of endothelial cell migration; blood coagulation, intrinsic pathway; negative regulation of fibrinolysis; negative regulation of angiogenesis; positive regulation of lipoprotein lipase activity |

* Gene symbols according to the UniProt database.

**Table S2 The results table of protein-protein interaction analysis on selected differentially expressed proteins**

| **#node1** | **node2** | **node1_string_id** | **node2_string_id** | **homology** | **coexpression** | **experimentally_determined_interaction** | **database_ annotated** | **automated_ textmining** | **Combined _score** |
| --- | --- | --- | --- | --- | --- | --- | --- | --- | --- |
| ACTA1 | VCL | 9606.ENSP00000355645 | 9606.ENSP00000211998 | 0 | 0.063 | 0.841 | 0.9 | 0.6 | 0.993 |
| ACTA1 | VASP | 9606.ENSP00000355645 | 9606.ENSP00000245932 | 0 | 0.062 | 0.213 | 0 | 0.988 | 0.991 |
| ACTA1 | DAG1 | 9606.ENSP00000355645 | 9606.ENSP00000442600 | 0 | 0 | 0 | 0.9 | 0.178 | 0.914 |
| ACTA1 | CFL1 | 9606.ENSP00000355645 | 9606.ENSP00000432660 | 0 | 0.065 | 0.947 | 0.9 | 0.636 | 0.997 |
| ACTA1 | GSN | 9606.ENSP00000355645 | 9606.ENSP00000362924 | 0 | 0.062 | 0.976 | 0.9 | 0.569 | 0.998 |
| APOA1 | GRN | 9606.ENSP00000236850 | 9606.ENSP00000053867 | 0 | 0 | 0 | 0 | 0.748 | 0.748 |
| APOA1 | APOH | 9606.ENSP00000236850 | 9606.ENSP00000205948 | 0 | 0.845 | 0.386 | 0.72 | 0.663 | 0.989 |
| APOA1 | GSN | 9606.ENSP00000236850 | 9606.ENSP00000362924 | 0 | 0 | 0 | 0.3 | 0.65 | 0.744 |
| APOA1 | PLG | 9606.ENSP00000236850 | 9606.ENSP00000308938 | 0 | 0.344 | 0 | 0 | 0.653 | 0.762 |
| APOA1 | FGB | 9606.ENSP00000236850 | 9606.ENSP00000306099 | 0 | 0.632 | 0.081 | 0 | 0.502 | 0.817 |
| APOA1 | SERPINC1 | 9606.ENSP00000236850 | 9606.ENSP00000356671 | 0 | 0.599 | 0.141 | 0 | 0.618 | 0.857 |
| APOA1 | FGG | 9606.ENSP00000236850 | 9606.ENSP00000336829 | 0 | 0.747 | 0.181 | 0 | 0.46 | 0.878 |
| APOA1 | F2 | 9606.ENSP00000236850 | 9606.ENSP00000308541 | 0 | 0.801 | 0 | 0 | 0.519 | 0.9 |
| APOA1 | FGA | 9606.ENSP00000236850 | 9606.ENSP00000306361 | 0 | 0.643 | 0.521 | 0.3 | 0.509 | 0.933 |
| APOA1 | LPL | 9606.ENSP00000236850 | 9606.ENSP00000309757 | 0 | 0 | 0 | 0.9 | 0.809 | 0.98 |
| APOA1 | APOC2 | 9606.ENSP00000236850 | 9606.ENSP00000466775 | 0 | 0.756 | 0 | 0.8 | 0.866 | 0.992 |
| APOC2 | APOH | 9606.ENSP00000466775 | 9606.ENSP00000205948 | 0 | 0.134 | 0 | 0.72 | 0.556 | 0.882 |
| APOC2 | LPL | 9606.ENSP00000466775 | 9606.ENSP00000309757 | 0 | 0 | 0 | 0.9 | 0.988 | 0.998 |
| APOH | LPL | 9606.ENSP00000205948 | 9606.ENSP00000309757 | 0 | 0 | 0 | 0.8 | 0.282 | 0.85 |
| APOH | PLG | 9606.ENSP00000205948 | 9606.ENSP00000308938 | 0 | 0.187 | 0.472 | 0 | 0.694 | 0.857 |
| APOH | FGA | 9606.ENSP00000205948 | 9606.ENSP00000306361 | 0 | 0.668 | 0.158 | 0 | 0.539 | 0.86 |
| APOH | FGB | 9606.ENSP00000205948 | 9606.ENSP00000306099 | 0 | 0.707 | 0.158 | 0 | 0.518 | 0.87 |
| APOH | SERPINC1 | 9606.ENSP00000205948 | 9606.ENSP00000356671 | 0 | 0.819 | 0.057 | 0 | 0.71 | 0.946 |
| APOH | FGG | 9606.ENSP00000205948 | 9606.ENSP00000336829 | 0 | 0.898 | 0.158 | 0 | 0.478 | 0.951 |
| APOH | F2 | 9606.ENSP00000205948 | 9606.ENSP00000308541 | 0 | 0.847 | 0 | 0 | 0.845 | 0.975 |
| C3 | MASP2 | 9606.ENSP00000245907 | 9606.ENSP00000383690 | 0 | 0.084 | 0.255 | 0 | 0.717 | 0.79 |
| C3 | MBL2 | 9606.ENSP00000245907 | 9606.ENSP00000363079 | 0 | 0.109 | 0 | 0 | 0.803 | 0.817 |
| C3 | MASP1 | 9606.ENSP00000245907 | 9606.ENSP00000296280 | 0 | 0.086 | 0.388 | 0 | 0.861 | 0.915 |
| C3 | PLG | 9606.ENSP00000245907 | 9606.ENSP00000308938 | 0 | 0.147 | 0.156 | 0.8 | 0.696 | 0.95 |
| C3 | CFHR4 | 9606.ENSP00000245907 | 9606.ENSP00000356386 | 0 | 0 | 0.834 | 0.6 | 0.97 | 0.997 |
| C3 | CFHR1 | 9606.ENSP00000245907 | 9606.ENSP00000314299 | 0 | 0 | 0.973 | 0.6 | 0.955 | 0.999 |
| CFL1 | VASP | 9606.ENSP00000432660 | 9606.ENSP00000245932 | 0 | 0.088 | 0 | 0 | 0.702 | 0.717 |
| CFL1 | GSN | 9606.ENSP00000432660 | 9606.ENSP00000362924 | 0 | 0.062 | 0 | 0 | 0.775 | 0.78 |
| DAG1 | HSPG2 | 9606.ENSP00000442600 | 9606.ENSP00000363827 | 0 | 0.107 | 0.705 | 0.8 | 0.991 | 0.999 |
| F2 | FGB | 9606.ENSP00000308541 | 9606.ENSP00000306099 | 0 | 0.612 | 0.803 | 0.9 | 0.666 | 0.997 |
| F2 | FGA | 9606.ENSP00000308541 | 9606.ENSP00000306361 | 0 | 0.801 | 0.96 | 0.9 | 0.621 | 0.999 |
| F2 | FN1 | 9606.ENSP00000308541 | 9606.ENSP00000346839 | 0 | 0.076 | 0.27 | 0 | 0.743 | 0.811 |
| F2 | FGG | 9606.ENSP00000308541 | 9606.ENSP00000336829 | 0 | 0.81 | 0.803 | 0.9 | 0.668 | 0.998 |
| F2 | SERPINC1 | 9606.ENSP00000308541 | 9606.ENSP00000356671 | 0 | 0.813 | 0.937 | 0.9 | 0.968 | 0.999 |
| FGA | HRG | 9606.ENSP00000306361 | 9606.ENSP00000232003 | 0 | 0.798 | 0.304 | 0 | 0.487 | 0.921 |
| FGA | FGB | 9606.ENSP00000306361 | 9606.ENSP00000306099 | 0.744 | 0.991 | 0.986 | 0.9 | 0.961 | 0.999 |
| FGA | PLG | 9606.ENSP00000306361 | 9606.ENSP00000308938 | 0 | 0.653 | 0.233 | 0 | 0.444 | 0.839 |
| FGA | FN1 | 9606.ENSP00000306361 | 9606.ENSP00000346839 | 0 | 0.088 | 0.444 | 0.72 | 0.353 | 0.895 |
| FGA | SERPINC1 | 9606.ENSP00000306361 | 9606.ENSP00000356671 | 0 | 0.925 | 0.259 | 0.9 | 0.543 | 0.997 |
| FGA | FGG | 9606.ENSP00000306361 | 9606.ENSP00000336829 | 0.764 | 0.993 | 0.986 | 0.9 | 0.961 | 0.999 |
| FGB | HRG | 9606.ENSP00000306099 | 9606.ENSP00000232003 | 0 | 0.65 | 0.086 | 0 | 0.54 | 0.839 |
| FGB | PLG | 9606.ENSP00000306099 | 9606.ENSP00000308938 | 0 | 0.662 | 0.251 | 0 | 0.43 | 0.843 |
| FGB | FN1 | 9606.ENSP00000306099 | 9606.ENSP00000346839 | 0 | 0.083 | 0.449 | 0.72 | 0.31 | 0.889 |
| FGB | SERPINC1 | 9606.ENSP00000306099 | 9606.ENSP00000356671 | 0 | 0.832 | 0.392 | 0.9 | 0.517 | 0.994 |
| FGB | FGG | 9606.ENSP00000306099 | 9606.ENSP00000336829 | 0.803 | 0.992 | 0.963 | 0.9 | 0.963 | 0.999 |
| FGF2 | FN1 | 9606.ENSP00000264498 | 9606.ENSP00000346839 | 0 | 0.088 | 0 | 0 | 0.925 | 0.93 |
| FGF2 | HSPG2 | 9606.ENSP00000264498 | 9606.ENSP00000363827 | 0 | 0.065 | 0.477 | 0.6 | 0.988 | 0.997 |
| FGG | HRG | 9606.ENSP00000336829 | 9606.ENSP00000232003 | 0 | 0.653 | 0.086 | 0 | 0.539 | 0.841 |
| FGG | PLG | 9606.ENSP00000336829 | 9606.ENSP00000308938 | 0 | 0.654 | 0.322 | 0 | 0.424 | 0.853 |
| FGG | FN1 | 9606.ENSP00000336829 | 9606.ENSP00000346839 | 0 | 0.088 | 0.449 | 0.72 | 0.308 | 0.889 |
| FGG | SERPINC1 | 9606.ENSP00000336829 | 9606.ENSP00000356671 | 0 | 0.91 | 0.392 | 0.9 | 0.594 | 0.997 |
| FN1 | VCL | 9606.ENSP00000346839 | 9606.ENSP00000211998 | 0 | 0.086 | 0.074 | 0.5 | 0.966 | 0.984 |
| FN1 | NID1 | 9606.ENSP00000346839 | 9606.ENSP00000264187 | 0 | 0.217 | 0 | 0 | 0.878 | 0.901 |
| FN1 | PLG | 9606.ENSP00000346839 | 9606.ENSP00000308938 | 0 | 0 | 0.27 | 0 | 0.989 | 0.992 |
| FN1 | GSN | 9606.ENSP00000346839 | 9606.ENSP00000362924 | 0 | 0.07 | 0.486 | 0 | 0.889 | 0.942 |
| FN1 | HSPG2 | 9606.ENSP00000346839 | 9606.ENSP00000363827 | 0 | 0.199 | 0.277 | 0 | 0.986 | 0.991 |
| GRN | HSPG2 | 9606.ENSP00000053867 | 9606.ENSP00000363827 | 0 | 0.065 | 0.486 | 0 | 0.986 | 0.992 |
| GSN | VCL | 9606.ENSP00000362924 | 9606.ENSP00000211998 | 0 | 0.094 | 0.27 | 0 | 0.772 | 0.836 |
| HRG | MBL2 | 9606.ENSP00000232003 | 9606.ENSP00000363079 | 0 | 0.619 | 0 | 0 | 0.252 | 0.703 |
| HRG | SERPINC1 | 9606.ENSP00000232003 | 9606.ENSP00000356671 | 0 | 0.667 | 0 | 0 | 0.803 | 0.931 |
| HRG | PLG | 9606.ENSP00000232003 | 9606.ENSP00000308938 | 0 | 0.422 | 0.225 | 0.8 | 0.992 | 0.999 |
| HSPG2 | VCL | 9606.ENSP00000363827 | 9606.ENSP00000211998 | 0 | 0.197 | 0 | 0 | 0.691 | 0.741 |
| HSPG2 | NID2 | 9606.ENSP00000363827 | 9606.ENSP00000216286 | 0 | 0.196 | 0.792 | 0.6 | 0.897 | 0.992 |
| HSPG2 | NID1 | 9606.ENSP00000363827 | 9606.ENSP00000264187 | 0 | 0.168 | 0.951 | 0.6 | 0.994 | 0.999 |
| HSPG2 | PLG | 9606.ENSP00000363827 | 9606.ENSP00000308938 | 0 | 0 | 0 | 0.9 | 0.386 | 0.936 |
| KRT14 | KRT5 | 9606.ENSP00000167586 | 9606.ENSP00000252242 | 0.781 | 0.742 | 0.972 | 0 | 0.989 | 0.994 |
| KRT2 | KRT5 | 9606.ENSP00000310861 | 9606.ENSP00000252242 | 0.958 | 0.09 | 0.837 | 0.3 | 0.599 | 0.89 |
| MASP1 | MASP2 | 9606.ENSP00000296280 | 9606.ENSP00000383690 | 0.923 | 0.066 | 0.27 | 0.9 | 0.99 | 0.931 |
| MASP1 | MBL2 | 9606.ENSP00000296280 | 9606.ENSP00000363079 | 0 | 0.14 | 0.875 | 0.9 | 0.97 | 0.999 |
| MASP2 | MBL2 | 9606.ENSP00000383690 | 9606.ENSP00000363079 | 0 | 0.14 | 0.874 | 0.9 | 0.963 | 0.999 |
| NID1 | NID2 | 9606.ENSP00000264187 | 9606.ENSP00000216286 | 0.913 | 0.288 | 0 | 0.65 | 0.808 | 0.758 |
| PLG | SERPINC1 | 9606.ENSP00000308938 | 9606.ENSP00000356671 | 0 | 0.674 | 0.391 | 0 | 0.917 | 0.982 |
| VASP | VCL | 9606.ENSP00000245932 | 9606.ENSP00000211998 | 0 | 0.062 | 0.494 | 0 | 0.989 | 0.994 |

**Table S3 Multivariate logistic regression analysis on the association of maternal plasma CFHR4 with fetal VSD**

| Variables | Coefficients | Z Value | OR (95% CI) | *P* value |
| --- | --- | --- | --- | --- |
| CFHR4* | 0.76 | 2.49 | 2.13 (1.18, 3.86) | 0.013 |
| Maternal BMI |  |  |  |  |
| 18.5-23.9 | ref | - | 1 |  |
| <18.5 | 2.46 | 1.77 | 11.73 (0.76, 180.17) | 0.077 |
| ≥ 24 | 2.19 | 2.51 | 8.96 (1.62, 49.55) | 0.012 |
| History of adverse pregnancy outcomes |  |  |  |  |
| No | ref | - | 1 | - |
| Yes | 1.46 | 2.11 | 4.31 (1.11, 16.78) | 0.035 |
| Periconceptional folic acid supplementation# |  |  |  |  |
| No | ref | - | 1 | - |
| Yes | 0.74 | 1.05 | 2.10 (0.53, 8. 8.37) | 0.29 |

* Included in as continuous variable and the corresponding OR was calculated for 100ng/ml increase.

# Defined as regular use of 400μg/day in the period from 3 months prior to pregnancy through the first trimester for lasting 12 weeks or more.
