# Supplementary figures and images for: Maternal serum CFHR4 protein as a potential non-invasive marker of ventricular septal defects in offspring: evidence from a comparative proteomics study
Source: Clin Proteomics. 2022 May 19;19:17. doi: 10.1186/s12014-022-09356-y (PMC9117979; doi:10.1186/s12014-022-09356-y)

**
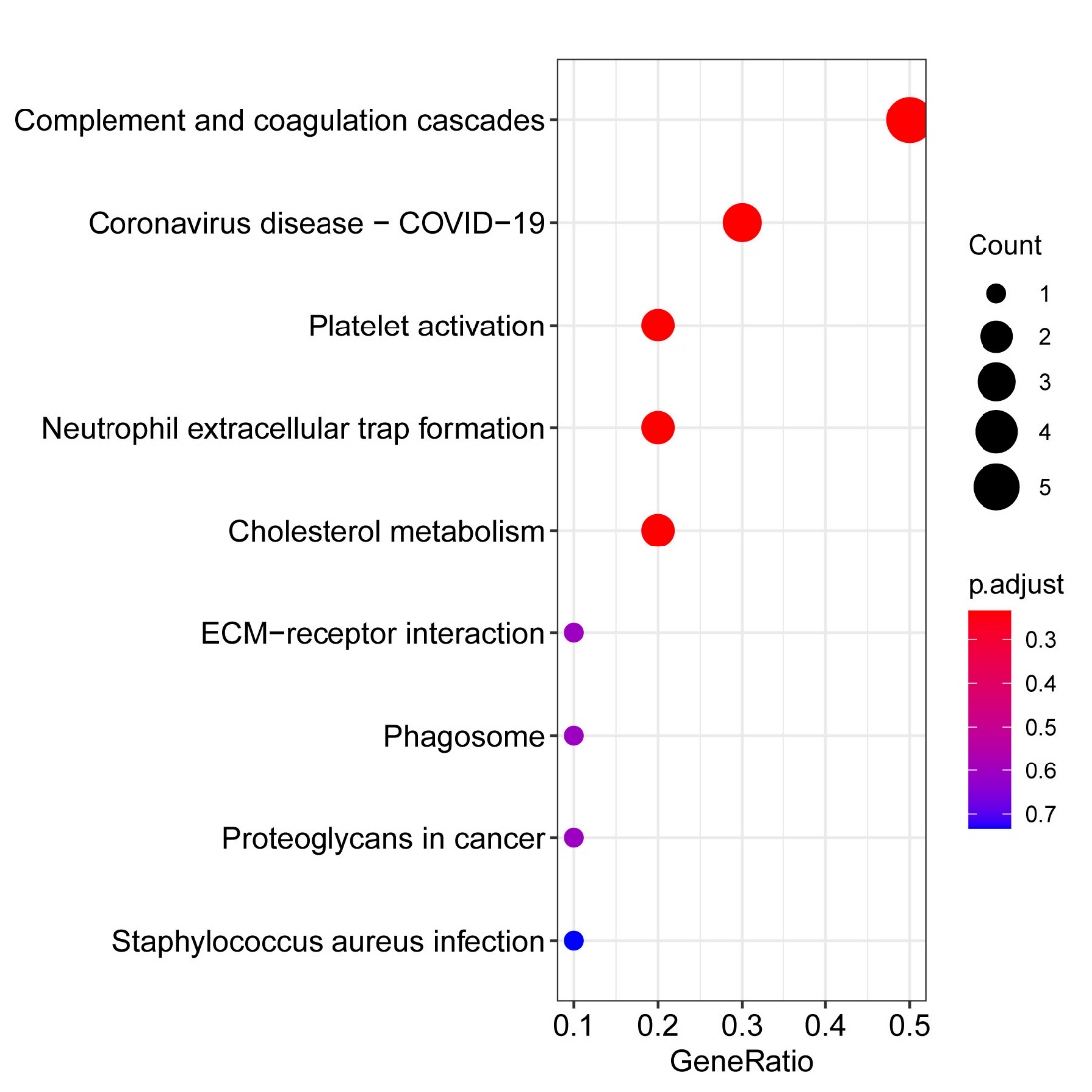
**

**Figure S1 KEGG pathway enrichment bubble plot of Differentially expressed proteins**

Supplement: Supplementary file 2 — Additional file 2: Fig. S1. KEGG pathway enrichment bubble plot of differentially expressed proteins. [file 12014_2022_9356_MOESM2_ESM.docx]
