# Supplementary material for: Maternal serum CFHR4 protein as a potential non-invasive marker of ventricular septal defects in offspring: evidence from a comparative proteomics study
Source: Clin Proteomics. 2022 May 19;19:17. doi: 10.1186/s12014-022-09356-y (PMC9117979; doi:10.1186/s12014-022-09356-y)
Supplement: Supplementary file 3 — Additional file 3: Fig. S2. The protein–protein interaction network of selected differentially expressed proteins. [file 12014_2022_9356_MOESM3_ESM.docx]

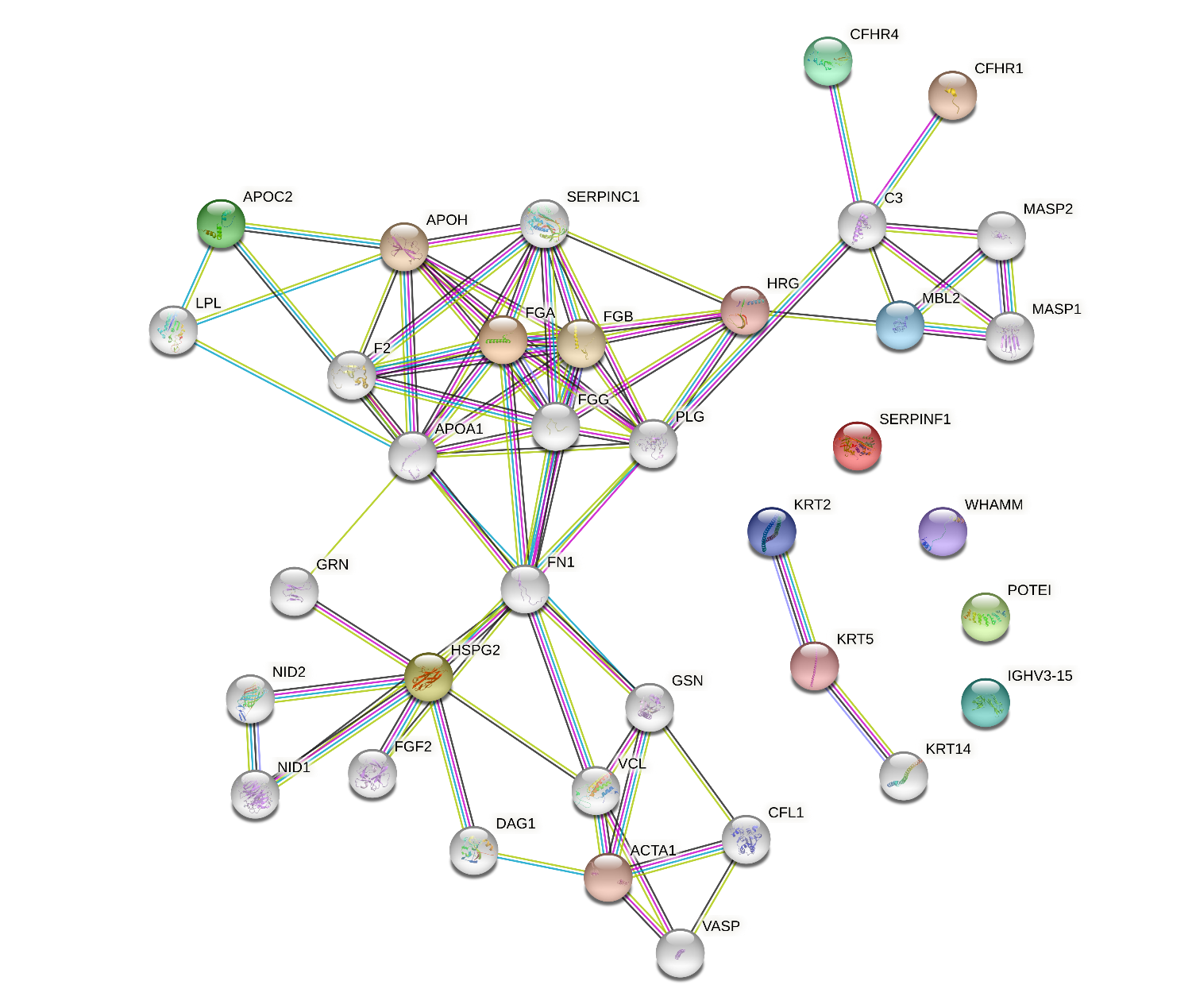


**Figure S2 The protein-protein interaction network of selected differentially expressed proteins.**

Colored nodes represent the query proteins and the first shell of interactors, while white nodes represent the second shell of interactors. Notably, the max number of interactors shown in the first and second shells were set to “query proteins only” and “no more than 20”, respectively.

Empty nodes indicate proteins of unknown 3D structure, filled nodes mean that some 3D structure is known or predicted.

Edges represent protein-protein associations.

Line color indicates the type of interaction evidence: light sea green = interactions from curated databases, purple = experimentally determined interactions, green = interactions predicted from gene neighborhood, red = interactions predicted from gene fusions, blue = interactions predicted from co-occurrence, yellow green = interactions based on text mining, dark = interactions from co-expression evidence, and corn flower blue = protein homology.
